# The impact of endometriosis on the outcome of Assisted Reproductive Technology

**DOI:** 10.1186/s12958-016-0217-2

**Published:** 2017-01-24

**Authors:** Mireia González-Comadran, Juan Enrique Schwarze, Fernando Zegers-Hochschild, Maria do Carmo B. Souza, Ramon Carreras, Miguel Ángel Checa

**Affiliations:** 1 Obstetrics and Gynecology, Hospital del Mar, Parc de Salut Mar de Barcelona, Barcelona, Spain; 2 GRI-BCN, Barcelona Infertility Research Group, Barcelona, Spain; 3 The Latin American Registry of ART, Montevideo, Uruguay; 4 Unit of Reproductive Medicine, Clínica Monteblanco, Santiago, Chile; 5 Unit of Reproductive Medicine, Clinica las Condes, and Program of Ethics and Public Policies in Human Reproduction University Diego Portales, Santiago, Chile; 6 Unit of Reproductive Medicine, Fertipraxis Centro de Reproducao Humana, Rio de Janeiro, Brazil; 7 Universitat Autònoma de Barcelona, Barcelona, Spain; 8 Passeig Marítim 25-29, 08003 Barcelona, Spain

**Keywords:** Endometriosis, Infertility, Assisted-reproductive technologies

## Abstract

**Background:**

Endometriosis has been described to impair fertility through various mechanisms. However, studies evaluating the reproductive outcomes of women undergoing assisted reproductive technologies show controversial results. The aim of this study is to assess whether the reproductive outcome is impaired among women with endometriosis-associated infertility undergoing IVF.

**Methods:**

A retrospective cohort study was performed, including women undergoing IVF reported by the *Red Latinoamericana de Reproduccion Asistida* (Redlara) registry, between January 2010 and December 2012. The study group included women with endometriosis-associated infertility, and the control group women with tubal factor, endocrine disorders or unexplained infertility. Women above 40 years, severe male factor and premature ovarian failure were excluded. The reproductive outcomes of between both groups were compared. The primary outcome was live birth. Secondary outcomes included clinical pregnancy, miscarriage, number of oocytes retrieved and number of fertilized oocytes. Outcomes were assessed after the first fresh IVF cycle, and were adjusted for age and number of embryos transferred.

**Results:**

A total of 22.416 women were included (3.583 with endometriosis and 18.833 in the control group). Mean age of patients in the endometriosis group and control group was 34.86 (3.47) and 34.61 (3.91) respectively, *p* = 0.000. The mean number of oocytes retrieved were 8.89 (6.23) and 9.86 (7.02) respectively, *p* = 0.000. No significant differences were observed between groups in terms of live birth (odds ratio (OR) 1.032, *p* = 0.556), clinical pregnancy (OR 1.044, *p* = 0.428) and miscarriage rates (OR 1.049, *p* = 0.623). Women with endometriosis had significantly lower number of oocytes retrieved (incidence risk ratio (IRR) 0.917, 95% CI 0.895–0.940), however, the number of fertilized oocytes did not differ among the two groups when adjusting for the number of oocytes retrieved (IRR 1.003, *p* = 0.794). An age-stratified analysis was performed, and no differences were observed in the reproductive outcomes between groups for women aged under 35 and 35 to 40.

**Conclusions:**

Reproductive outcomes among women undergoing IVF and diagnosed with endometriosis-associated infertility do not differ significantly from women without the disease. Although women with endometriosis generate fewer oocytes, fertilization rate is not impaired and the likelihood of achieving a live birth is also not affected.

## Background

Endometriosis affects between 0.5 and 10% of women in reproductive age [1–3] and approximately 25–40% of women diagnosed with infertility [1]. Endometriosis is defined as the presence of endometrial tissue outside the uterine cavity [4]. This ectopic tissue induces a chronic inflammatory reaction that may lead to extensive fibrosis and adhesion formation. Clinically, endometriosis is classified as peritoneal, ovarian, and deep-infiltrating, although they often coexist [5].

Depending on the location of the endometriotic implant and the severity of the disease, several mechanisms have been postulated to explain the reduced fecundity observed in women affected by this condition. To date, the evidence suggests that endometriosis has deleterious effects on ovarian function [6, 7], tubal function [8], and may also affect endometrial receptivity [9]. Abnormal folliculogenesis and oocyte maturation [6, 7], increased radical oxidative stress [10, 11], as well as imbalances in the levels of cytokines, interleukins and various growth factors have been described as potential contributors to impaired fertility [11, 12].

There is a serious controversy as to whether endometriosis per se, can affect the reproductive outcomes of women undergoing assisted reproductive technologies (ART). Several authors have found that endometriosis have a negative impact [13–15], while others have not found such association [16–20].

The aim of this study is to compare, in a large cohort of women undergoing ART, the reproductive outcome of women with endometriosis with that of women without endometriosis.

## Methods

### Study design

A retrospective cohort study was performed from a prospectively collected database by the Latin American registry of Assisted Reproduction between January 2010 and December 2012. This database corresponds to an individualized case-by-case registry that keeps record of data of every case performed by 145 centers belonging to The Latin America Network of Assisted Reproduction (REDLARA).

In order to be certified by REDLARA, the consent forms signed by patients must include a statement stating that the data collected may be published in epidemiological studies, which will keep anonymity. When desired, patients can ask for their data to be removed of the database. Therefore no Institutional Review Board/Ethics Committee was sought.

In addition, STROBE (STrengthening the Reporting of OBservational studies in Epidemiology) guidelines for cohort studies were followed to conduct this study [21]. The STROBE statement consist of a 22-item checklist which refers to the abstract, introduction, methodology, results and discussion of observational studies, and provide a guideline for authors intended to make strong contributions to improving quality of reporting of these observational studies.

### Selection of participants and data collection

Women under the age of 40 years old undergoing ART were included in the study. The study group consisted of women diagnosed with infertility associated with endometriosis. The control group included women with tubal factor, endocrine disorders or unexplained infertility. The diagnosis of endometriosis was performed in the past and reported by the institution.

Cases with women aged ≥ 40, premature ovarian failure, severe male factor, and women undergoing IVF for preimplantation genetic diagnosis were excluded from this study.

Baseline characteristics were recorded prospectively, as well as data from the infertility evaluation,and outcomes derived from the controlled ovarian stimulation including: number of oocytes retrieved, fertilization rate, as well as pregnancy and live birth rates.

The primary outcome was live births. Secondary outcomes included clinical pregnancy, miscarriage, number of retrieved oocytes, and number of fertilized oocytes. Outcomes were assessed after the first fresh IVF cycle and analyzed per initiated cycle. These outcomes were defined according to the terminology recommended in the ICMART-WHO (International Committee Monitoring Assisted Reproductive Technologies, World Health Organization) glossary [22] and the updated and revised nomenclature for the description of early pregnancy events [23].

The database used to enter patients information by Redlara centers had an internal validation program which checks for consistency and does not allow inconsistent data from one set to another. Furthermore, each participating institution is certified before their data is accepted in the registry.

### Statistical analysis

Descriptive data of the study population are presented as percentages for categorical variables, and as mean (± standard deviation) for numeric variables. Data from both, study and control groups, were compared using Fisher’s exact test for categorical variables and student’s t-test for discrete numerical variables. Outcomes were analyzed using logistic regression models for categorical variables and negative binominal regression models for discrete numerical variables, adjusting for age of the female partner and number of embryos transferred. The analysis of the number of fertilized oocytes was additionally adjusted for the number of oocytes retrieved. *P*-values below 0.05 were considered as statistically significant. Reproductive outcomes were expressed as odds ratio (OR) or incidence risk ratio (IRR) when using logistic regression models or negative binomial regression models, respectively. An age-stratified analysis was performed of the reproductive outcomes for women aged under 35 and 35 to 40.

We estimated that the sample size of the cohort was sufficient to detect a 3% difference in livebirth rate between groups, with a type I error of 0.05 and a power of 90%.

## Results

A total of 22,416 women were included in the study, 3583 women with the diagnosis of endometriosis and 18,833 women without the diagnosis of endometriosis. In the control group 36.66% had been diagnosed with tubal factor, 20.02% with endocrine disorders, 40.66% with unexplained infertility and in 2.48% of cases the diagnosis was not available. Mean age was 34.83 ± 3.47 years in the endometriosis group and 34.61 ± 3.91 in the control group (*p* < 0.001). This difference in 0.22 years is not considered clinically significant.

A descriptive analysis of the results obtained in the fresh IVF/ICSI cycle is shown in Table 1. Women diagnosed with endometriosis had fewer oocytes retrieved as compared with the control group, 8.89 (6.23) versus 9.86 (7.02) (*p* < 0.001), respectively, differences that were also observed in the age-stratified analysis (Table 2). Cancellation rate was also significantly higher in the endometriosis group compared to the control group, 2.95% versus 3.94% (p0.005), respectively, differences that remained statistically significant in the age-stratified analysis. However, the fertilization rate was similar between both groups, 60.28% versus 59.54%, respectively, *p* = 0.159.

**Table 1:** Descriptive data of the study population and results of the IVF cycle

	Control (*n* = 18,833)	Endometriosis (*n* = 3,583)	*P* values
Age	34.61 ± 3.91	34.83 ± 3.47	<0.001
N° oocytes retrieved	9.86 ± 7.02	8.89 ± 6.23	<0.001
Cancellation rate	2.95%	3.94%	0.005
Fertilization rate	59.54%	60.28%	0.159
Number of embryos transferred per ET	2.22 ± 0.70	2.15 ± 0.65	<0.001
Clinical pregnancy rate	23.76%	24.31%	0.483
Miscarriage rate	5.88%	5.86%	0.975
Live birth rate	23.35%	23.81%	0.563

*Note:* Values presented as mean ± SD or %

**Table 2:** Descriptive data of the study population and results of the IVF cycle according to woman’s age category

	Control	Endometriosis	*P* values
Age < 35 years	*n* = 8,454	*n* = 1,516	
Age	31.04 ± 2.73	31.50 ± 2.35	<0.001
N° oocytes retrieved	11.64 ± 7.48	10.32 ± 6.56	<0.001
Cancellation rate	2.12%	2.97%	0.0066
Fertilization rate	57.51%	60.10%	0.002
Number of embryos transferred per ET	2.16 ± 0.61	2.10 ± 0.57	<0.002
Clinical pregnancy rate	27.52%	30.14%	0.040
Miscarriage rate	6.06%	5.58%	0.463
Live birth rate	27.08%	29.48%	0.058
Age 35–40 years	*n* = 10,379	*n* = 2,067	
Age	37.51 ± 1.70	37.27 ± 1.65	<0.001
N° oocytes retrieved	8.39 ± 6.24	7.82 ± 5.74	<0.001
Cancellation rate	3.63%	4.64%	0.004
Fertilization rate	61.24%	60.41%	0.204
Number of embryos transferred per ET	2.27 ± 0.76	2.18 ± 0.70	<0.001
Clinical pregnancy rate	20.70%	20.02%	0.490
Miscarriage rate	5.73%	6.07%	0.555
Live birth rate	20.32%	19.64%	0.500

*Note:* Values presented as mean ± SD or %

### Reproductive outcomes

In the multivariate analysis of the reproductive outcomes, after adjusting for the age of the female partner and the number of embryos transferred, there was no significant difference in live birth between the two groups (OR 1.032, 95% CI 0.927 – 1.151). Furthermore, no differences were found in clinical pregnancy rate (OR 1.044, 95% CI 0.938–1.162) and miscarriage rate (OR 1.048, 95% CI 0.867–1.269).

Interestingly, although in the group of women with endometriosis the number of oocytes retrieved was significantly lower (IRR 0.917, 95% CI 0.895–0.940), the number of fertilized oocytes did not differ (IRR 1.003, 0.983 – 1.023) after adjusting for the number of oocytes retrieved.

Additionally, there was a higher cancellation rate in the endometriosis group as compared to the control group, 3.94% versus 2.95%, although this difference in 0.99% was not considered clinically significant.

**Fig. 1:**
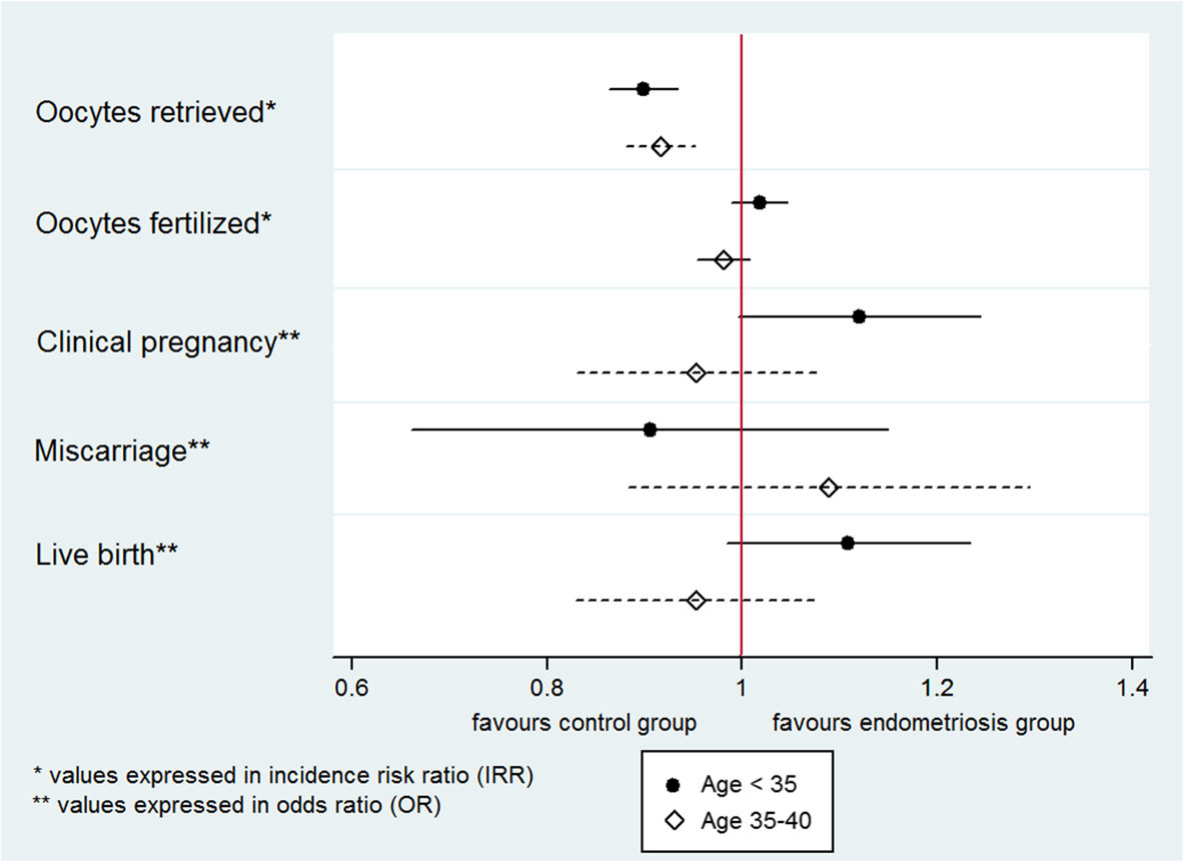
Reproductive outcomes of women with endometriosis compared to the control group, age-stratified analysis

In the age-stratified analysis, the odds ratios for these reproductive outcomes were consistent with these previous results. Women aged under 35 with endometriosis showed no differences in live birth (OR 1.11, 95% CI 0.984 – 1.263), clinical pregnancy (OR 1.127, 95% CI 0.996 – 1.276) and miscarriage rates (OR 0.910, 95% CI 0.713 – 1.161), compared to women in the control group, Fig. 1. In this group, women with endometriosis also had significantly lower number of retrieved oocytes (IRR 0.903, 95% CI 0.872 – 0.936), although fertilization rates were comparable after adjusting for the number of oocytes retrieved (IRR 1.018, 95% CI 0.989 – 1.048).

Among women aged between 35 and 40, live birth (OR 0.953, 95% CI 0.842 – 1.078), clinical pregnancy (OR 0.954, 95% CI 0.844 – 1.078) and miscarriage rates (OR 1.093, 5% CI 0.890 – 1.342) were also comparable between the endometriosis and the control group. Besides, women with endometriosis also retrieved a statistically lower number of oocytes retrieved (IRR 0.920, 95% CI 0.888 – 0.952). However, after adjusting for this variable, fertilization rates were comparable (IRR 0.981, 95% CI 0.955 – 1.007), Fig. 1.

## Discussion

The main finding of this study show that endometriosis does not have a significant impact in live birth rate among infertile women undergoing IVF/ICSI.

Previous reports on the outcome of ART for endometriosis-associated infertility have showed conflicting results. Barnhart et al. [24] reported in a systematic review and meta-analysis a significant decrease in pregnancy rates among women with endometriosis compared with women with tubal factor. In addition, they described that women with endometriosis showed a significant reduction in the number of oocytes retrieved, as well as lower fertilization and implantation rates.

Interestingly, a more recent meta-analysis by Harb et al. [25] described that ART results were dependent on the severity of the disease. The presence of severe endometriosis was associated with reduced implantation and clinical pregnancy rates, although the reduction in live birth rate did not reach statistical significance. Meanwhile, women with mild endometriosis showed comparable results in terms of implantation, clinical pregnancy and live birth rates [25]. In contrast, the review by Barbosa et al. [26] did not report significant differences in live birth and clinical pregnancy rates among women with endometriosis as compared with other causes of infertility. Furthermore, reproductive outcomes were not affected by the severity of endometriosis.

When evaluating the effect of ovarian endometriosis on the response to controlled ovarian stimulation (COS) for ART, the available literature show inconsistent results [16–18, 27, 28]. Ballester et al. [27] reported comparable ART results when comparing women with isolated endometriomas and women with coexisting ovarian and deep-infiltrating endometriosis. Meanwhile, a recent study by Ashrafi et al. [17] observed in a prospective cohort a significantly poorer ovarian response to stimulation and lower number of metaphase-II oocytes retrieved among women with endometriomas as compared with a control group. Nevertheless, the quality of the embryos obtained and clinical pregnancy rates were comparable.

In term of implantation, various studies have reported an impairment in endometrial decidualization and receptivity, including a local dysregulation in the progesterone response, an alteration in the expression of integrins and interleukins among others [29–31]. However, studies examining embryo implantation, show contradicting results [32–34]. In a retrospective analysis of ART outcomes and oocyte donation programs, Simón et al. [33], reported a significantly reduced pregnancy and implantation rates when oocytes came from donors with endometriosis, although implantation rates among women with and without endometriosis were comparable when oocytes came from donors without endometriosis. These results suggest that infertility in patients with endometriosis seem to be more dependent on the quality of the oocyte rather than an impaired decidualization.

Overall, we hypothesize that the discrepancies among different studies are explained by the disparity_when controlling for confounding factors due to the nature of the different study design, as well as the high heterogeneity of the inclusion criteria and the low number of patients enrolled.

When comparing published literature with the results obtained in our study, even though women with endometriosis-associated infertility yielded significantly lower number of oocytes, fertilization rates were comparable with that of the control group (Fig. 1). Besides, after adjusting for the number of embryos transferred and the age of the female partner, clinical pregnancy, miscarriage and live birth rates did not differ in the two groups. In a further attempt to assess whether this lack of difference was affected by age, the odds ratios obtained in the age-stratified analysis did not vary when comparing women with endometriosis and the control group neither under the age of 35 nor between the ages of 35 and 40 (Fig. 1), suggesting that these outcomes were not influenced significantly by the loss of ovarian reserve.

There are several factors that are consistent with our analysis. Most significantly, the large number of patients included in the study, the largest cohort published so far [26], which provided a considerable gain in statistical power. Besides, the prospective nature of the database in addition to its individualized case-by-case registry empower the results obtained in the analysis. Furthermore, data from this study is obtained from a multinational database, and compared to a national database, it encompasses a wider variety of ethnics, enhancing the external validity of our analysis.

However, this study has several limitations that need to be addressed. First, it was not possible to differentiate mild from moderate or severe endometriosis in the analysis, since these data were not available in the registry. In addition, no distinction was made between women who had undergone ovarian surgery to remove endometriotic lesions prior to ART from those who had not, impeding the identification of patients with a potentially reduced ovarian response to hyperstimulation. Furthermore, it was not possible to identify women who were disease-free at the time of IVF from those with active disease. Hence, a stratified analysis of patients with poorer prognosis, particularly in terms of live birth, was not possible.

## Conclusions

We conclude, that in spite of its limitations, the data examined in this study is a reflection that the rate of live birth after ART, is not affected by the presence or past history of endometriosis.

## Abbreviations

ART: Assisted Reproductive Technologies
CI: Confidence interval
COS: Controlled ovarian stimulation
ET: Embryo transfer
ICMART: International Committee Monitoring Assisted Reproductive Technologies
ICSI: Intra-cytoplasmatic Sperm Injection
IRR: Incidence risk ratio
IVF: In vitro fertilization
OR: Odds ratio
REDLARA: *Red Latinoamericana de Reproduccion Asistida* (Latin American Network of Assisted Reproduction)
STROBE: Strengthening the Reporting of Observational studies in Epidemiology
WHO: World Health Organization
